# Non-linear relationship between the body roundness index and incident type 2 diabetes in Japan: a secondary retrospective analysis

**DOI:** 10.1186/s12967-022-03321-x

**Published:** 2022-03-07

**Authors:** Liling Wu, Hailu Pu, Man Zhang, Haofei Hu, Qijun Wan

**Affiliations:** 1 Department of Nephrology, The First Affiliated Hospital of Shenzhen University, Shenzhen, 518000 Guangdong Province China; 2 Department of Nephrology, Shenzhen Second People’s Hospital, No. 3002 Sungang Road, Futian District, Shenzhen, 518000 Guangdong Province China; 3 Department of Functional Neurology, The First Affiliated Hospital of Shenzhen University, Shenzhen, 518000 Guangdong Province China; 4 Department of Functional Neurology, Shenzhen Second People’s Hospital, No. 3002 Sungang Road, Futian District, Shenzhen, 518000 Guangdong Province China

**Keywords:** Body roundness index, Incident type 2 diabetes mellitus, Non-linear relationship

## Abstract

**Background:**

Body roundness index (BRI) is one of the obesity-related anthropometric indices. However, studies on the relationship between BRI and diabetes risk is limited. The purpose of this study was to explore the relationship between baseline BRI and incident type 2 diabetes mellitus (T2DM) in the Japanese population.

**Methods:**

A retrospective longitudinal study of 15,310 participants in a physical examination program at Murakami Memorial Hospital in Japan from 2004 to 2015. The association between BRI levels and incident T2DM was analyzed by Cox proportional-hazards regression, smooth curve fitting, subgroup analyses, and a set of sensitivity analyses. Receiver operating characteristic curve analysis was used to assess the ability of BRI to predict diabetes.

**Result:**

Baseline BRI levels were elevated in participants who developed T2DM. Baseline BRI levels were positively associated with incident T2DM after adjusting confounding variables (HR = 1.570, 95% CI 1.360–1.811). Additionally, we did a set of sensitivity analyses to ensure the robustness of the results. There was also a non-linear relationship between BRI and incident diabetes in both genders, and the inflection point of BRI was 4.137 in females and 3.146 in males. We found a strong positive correlation between BRI and the incidence of diabetes on the right of the inflection point (Male: HR = 1.827, 95% CI 1.449–2.303; Female: HR = 4.189, 95% CI 1.862–9.421). What’s more, among the anthropometric indices, BRI showed the optimal capability to predict T2DM (Male: AUC = 0.706, 95% CI 0.674–0.738; Female: AUC = 0.735, 95% CI 0.676–0.795).

**Conclusion:**

An elevated BRI level in baseline was independently associated with incident T2DM. Baseline BRI improves the identification of patients at risk of T2DM and may enable early and optimized therapy to improve their outcomes.

**Supplementary Information:**

The online version contains supplementary material available at 10.1186/s12967-022-03321-x.

## Background

The global prevalence of diabetes among adults has been increasing in recent decades [1]. Diabetes brings enormous economic pressure on national health systems around the world, so prevention efforts are needed to reduce this burden [2]. Studies have shown that one of the main risk factors for type 2 diabetes (T2DM) is obesity. Elevated fatty acids in obesity and overweight impair insulin action, leading to insulin resistance and T2DM [3].

Body mass index (BMI) provides the convenient way to assess overweight and obesity, but it fails to distinguish between the accumulation of muscle and fat, leading muscular people to be misdiagnosed as overweight or obese [4]. Thomas et al. developed a anthropomorphic index body roundness index (BRI) to predictor body fat and visceral adipose tissue volume in 2013. It is calculated based on waist circumference (WC) and height [5]. Several cross-sectional studies have established the potential of BRI to identify T2DM. However, a large longitudinal study of BRI and T2DM in Japanese is limited. A non-linear relationship between T2DM and BRI has not been reported.

In the present study, we postulated that the baseline BRI level might serve as an early predictor of incident T2DM in Japan. To test this hypothesis, a retrospective longitudinal study was performed in participants who under physical examination program. The relationship between BRI levels and the risk of T2DM was explored in Japan. In addition, the relationship between BRI and incident T2DM was applied by generalized additive model (GAM). Our findings demonstrate that tracking the baseline BRI aids in the prediction of incident T2DM. This will help clinicians plan and initiate the management strategies early to improve outcomes for participants with prediabetes.

## Methods

### Study design

**Fig. 1:**
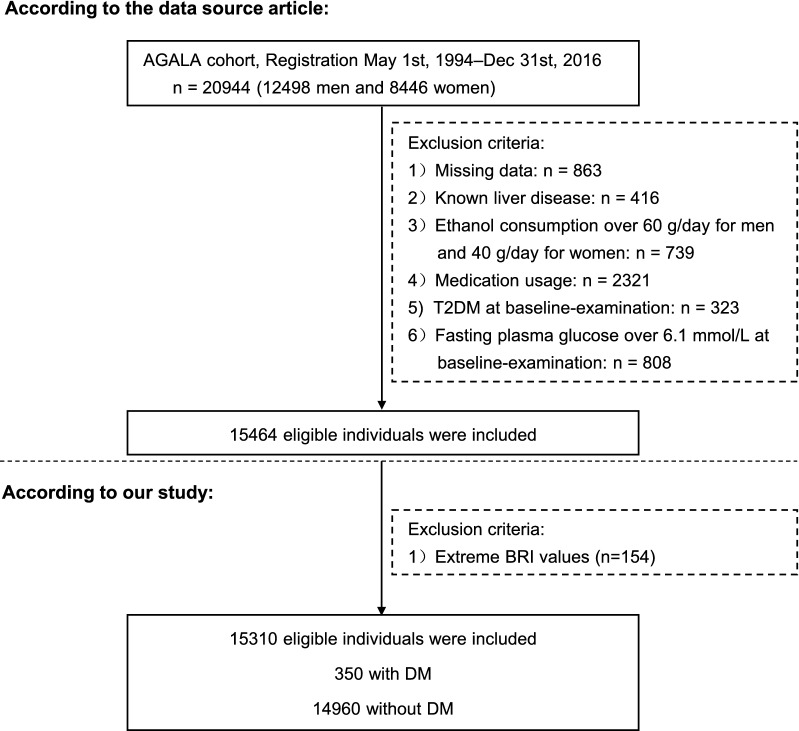
Study design and participant flow. *NAGALA* NAfld in Gifu Area, Longitudinal Analysis; *T2DM* type 2 diabetes mellitus

The study is a secondary retrospective study [6]. Data was obtained from the “DATADRYAD” (www. Datadryad.org) database. We quoted the Dryad data package on the basis of Dryad Terms of Service (Dryad data package: Takuro Okamura et al. (2018) Data from: Ectopic fat obesity presents the greatest risk for T2DM: a population-based longitudinal study. Dryad Digital Repository). Original data were obtained from NAGALA (NAfld in the Gifu Area, Longitudinal Analysis) database, which recruited 20,944 Japaneses who underwent a medical examination at Murakami Memorial Hospital from 2004 to 2015. Fifteen thousand four hundred and sixty-four participants in the analysis according to the following exclusion criteria: (1) participants diagnosed alcoholic fatty liver disease, viral hepatitis (n = 416), diabetes (n = 323) (2) anyone who took medication at baseline examination (n = 2321) (3) those with fasting plasma glucose ≥ 6.1 mmol/L (n = 808) (4) individuals who had a heavy drinking habit (ethanol consumption over 60 g/day for men and 40 g/day for women) (n = 739) (5) participants with missing covariate data were also excluded (n = 863). In addition, the anthropometric indices were calculated using the following equations: BMI = weight/height^2^, a body shape index (ABSI) = $\frac{{{\text{WC}}}}{{{\text{BMI}}^{2/3} {\text{height}}^{1/2} }}$, $${\text{BRI}} = 364.2 - 365.5 \times \sqrt {1 - \left( {\frac{{\begin{array}{*{20}c} {\left( {\frac{{{\text{WC}}}}{2\Pi }} \right)^{2} } \\ \end{array} }}{{(0.5\;\;{\text{height}})^{2} }}} \right)}$$. Individuals were excluded if the BRI value was an extreme value (the extreme value = mean ± 3SD) (n = 154) [7]. Finally, 15,310 subjects (8364 male and 6946 female) included in this study. Figure 1 described the study design and participant flow.

### Data collection

Variables contained in the database file were as follows: sex, age, diastolic blood pressure (DBP), systolic blood pressure (SBP), WC, fasting plasma glucose (FPG), hemoglobinA1c (HbA1c), high-density lipoprotein cholesterol (HDL-C), triglyceride (TG), total cholesterol (TC), ethanol consumption, gamma-glutamyltransferase (GGT), alanine aminotransferase (ALT), aspartate aminotransferase (AST), smoking status, fatty liver, exercise, days of follow-up and incident diabetes.

Lifestyle factors, including smoking and drinking habits, medical history, and physical activity, were obtained using questionnaires. Participants were divided into the following groups: no or minimal alcohol consumption: < 40 g/week; light alcohol consumption: 40–140 g/week; moderate alcohol consumption: 140–280 g/week; or heavy alcohol consumption: > 280 g/week [8]. The participants were categorized by smoking status: non-smokers were defined as never smokers, ex-smokers were past smokers who quit before the baseline visit, and current smokers were smokers at the baseline visit [6]. Regular exercisers are participants who regularly perform any type of exercise > 1 time/week [9]. Fatty liver was diagnosed by ultrasonography performed by trained technicians [10].

### Follow-up and outcome definitions

Diabetes was defined as fasting plasma glucose ≥ 7 mmol/L, HbA1c ≥ 6.5%, or self-reported during the follow-up period [11]. During follow up, the outcome was the incident T2DM. The participants who lost to follow-up would still be analyzed in the study.

### Statistical analysis

BRI was stratified into four groups: Q1 < 2.09; 2.09 ≤ Q2 < 2.63; 2.63 ≤ Q3 < 3.23; 3.23 ≤ Q4. Continuous variables with normal and skewed distributions were expressed as mean with standard deviation or median with interquartile range, and compared by one-way ANOVA or Kruskal–Wallis. Categorical variables were expressed as percentages of specific groups and compared by chi-square test. Cumulative incidence and person-year incidence were used to express incidence rate.

The Cox proportional hazards model was used to estimate the association between BRI and the incident T2DM with baseline BRI fitted as a continuous variable and categorical variables (stratified into 4 subgroups: Q1 < 2.09; 2.09 ≤ Q2 < 2.63; 2.63 ≤ Q3 < 3.23; 3.23 ≤ Q4).The lowest baseline BRI category was used as a common reference to compute the hazard ratios (HRs) and 95% confidence intervals (CIs) for the other baseline BRI strata. The results of unadjusted, minimally adjusted (age, gender, SBP, DBP, ethanol consumption, smoking status, and exercise) and fully adjusted (age, gender, SBP, DBP, FPG, ALT, AST, HbA1c, TC, HDL-C, ethanol consumption, smoking status, exercise, fatty liver) analyses are shown. The minimally adjusted was given as model I in Table 3 and the the fully adjusted was given as model II in Table 3. Survival estimates and time-to-event variables were computed using the Kaplan–Meier method. A log-rank test was used to compare the Kaplan–Meier probability of diabetes-free survival among BRI groups.

A series sensitivity analysis wer conducted to ensure the robustness of the relationship between BRI with the incident T2DM. The multivariable Cox proportional hazards model were repeated in the data set including participants without fatty liver or any alcohol consumption.

Applying smooth curve fitting and GAM to demonstrate the association of BRI with T2DM stratified by sex. The threshold effect of BRI on event T2DM was calculated using a two-piece linear regression model.

To assess the consistency of the association between BRI and the incident T2DM, we repeated the analyses in subgroups defined by sex, age, ethanol consumption, baseline SBP (< 140 mmHg,  ≥ 140 mmHg), HDL-C (< 1 mmol/L,  ≥ 1 mmol/L), ALT (< 40U/L,  ≥ 40U/L), baseline DBP(< 90 mmHg,  ≥ 90 mmHg), exercise, ethanol consumption, smoking status.

Receiver operating characteristic (ROC) curve was applied to estimate the ability of BRI, WC, ABSI and BMI to predict the risk of T2DM stratified by sex.

All of these analyses were performed with Empower-Stats (http://www.empowerstats.com, X&Y Solutions, Inc., Boston, MA) and the statistical software package R (http://www.R-project.org, The R Foundation). P values less than 0.05 (two-sided) were considered significant statistically.

### Ethics approval and consent to participate

The author of the original study waived all copyright and related privilege of these data. This study was approved by the Ethics Committee of the Murakami Memorial Hospital, and informed consent was obtained from all subjects.

## Result

### Demographics and characteristics of the participants

**Fig. 2:**
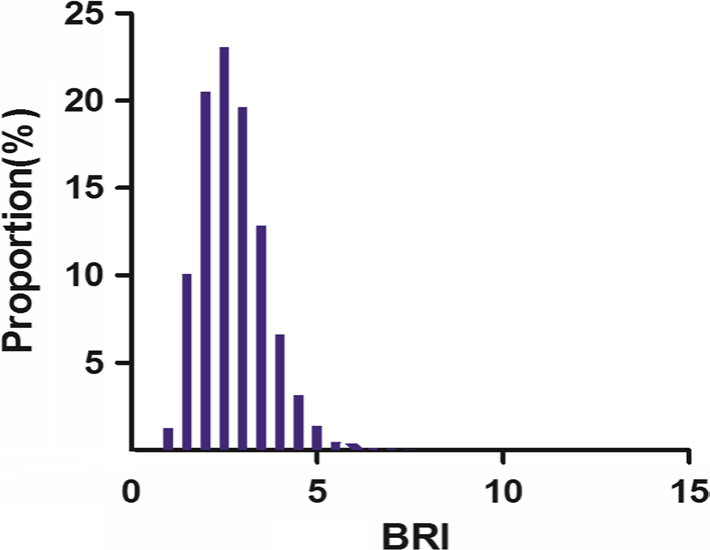
Distribution of BRI. It presented a skewed distribution while being in the range from 0.151 to 5.427

**Fig. 3:**
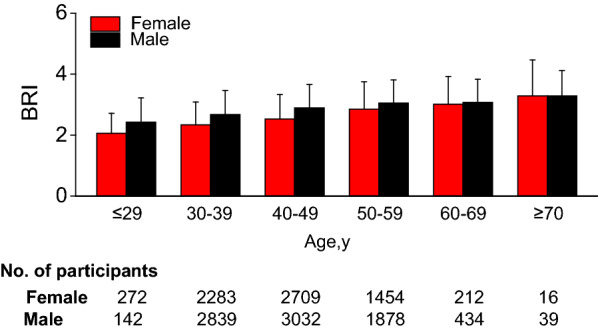
Bar graphs depicting mean BRI levels stratified according to age and gender categories

The study included 15310 participants. The mean follow-up time was 5.39 years, of which 350 participants developed T2DM during the follow-up period, as shown in Fig. 1. The BRI value was normally distributed, ranging from 0.151 to 5.427 (Fig. 2). In age stratification by 10 intervals, male subjects had a higher BRI values than female subjects no matter what age group they were in (Fig. 3). We also found that the BRI values increased with age, both in male and female participants.

The characteristics of the participants were stratified by the quartiles of the BRI. About the anthropometric indices, participants with the highest quartiles of the BRI had the highest age, WC, BMI, ethanol consumption (*P* < 0.001; Table 1). What's more, participants with the highest quartiles of the BRI had the highest FBG, HbA1c, TC,TG, GGT, ALT, AST, SBP, DBP,WC and BMI values. Besides, HDL-C is high in participants with the lowest BRI range. (*P* < 0.001; Table 1).

**Table 1:** The baseline characteristics of participants

BRI	Q1 (< 2.09)	Q2 (2.09–< 2.63)	Q3 (2.63–< 3.23)	Q4 (≥ 3.23)	*P* value
Participants	3828	3827	3827	3828	
Age, years	40.80 ± 8.39	42.67 ± 8.47	44.56 ± 8.54	46.75 ± 9.04	< 0.001
Gender—n (%)					< 0.001
Female	2414 (63.062%)	1790 (46.773%)	1406 (36.739%)	1336 (34.901%)	
Male	1414 (36.938%)	2037 (53.227%)	2421 (63.261%)	2492 (65.099%)	
Smoking status					< 0.001
Never-smoker	2675 (69.88%)	2284 (59.68%)	2007 (52.44%)	1962 (51.25%)	
Past-smoker	451 (11.78%)	697 (18.21%)	878 (22.94%)	912 (23.82%)	
Current-smoker	702 (18.34%)	846 (22.11%)	942 (24.61%)	954 (24.92%)	
Ethanol consumption g/wk	31.38 ± 63.84	48.00 ± 80.30	55.92 ± 89.75	56.57 ± 90.76	< 0.001
Habit of exercise	689 (18.00%)	758 (19.81%)	676 (17.66%)	573 (14.97%)	< 0.001
WC (m)	0.66 ± 0.05	0.73 ± 0.04	0.79 ± 0.04	0.86 ± 0.06	< 0.001
BMI (kg/m^2^)	19.02 ± 1.53	21.02 ± 1.52	22.69 ± 1.70	25.35 ± 2.45	< 0.001
FBG (mmol/L)	4.99 ± 0.39	5.11 ± 0.39	5.22 ± 0.40	5.32 ± 0.39	< 0.001
HbA1c(%)	5.104 ± 0.303	5.138 ± 0.308	5.177 ± 0.318	5.257 ± 0.334	< 0.001
TC (mmol/L)	4.87 ± 0.81	5.02 ± 0.83	5.20 ± 0.85	5.40 ± 0.85	< 0.001
TG (mmol/L)	0.53 (0.38–0.73)	0.65 (0.46–0.96)	0.84 (0.58–1.24)	1.05 (0.70–1.55)	< 0.001
HDL-C (mmol/L)	1.66 ± 0.40	1.527 ± 0.392	1.386 ± 0.381	1.281 ± 0.346	< 0.001
GGT (U/L)	14.41 ± 11.20	18.11 ± 15.27	21.98 ± 20.19	26.34 ± 21.24	< 0.001
ALT (U/L)	14.00 (11.00–18.00)	15.00 (12.00–20.00)	18.00 (13.00–24.00)	21.00 (16.00–31.00)	< 0.001
AST (U/L)	16.00 (13.00–19.00)	17.00 (14.00–20.00)	18.00 (14.00–21.00)	19.00 (15.00–24.00)	< 0.001
SBP (mmHg)	106.89 ± 12.80	111.91 ± 13.06	116.32 ± 13.91	122.08 ± 15.14	< 0.001
DBP (mmHg)	66.40 ± 8.82	69.62 ± 9.36	72.95 ± 10.05	76.88 ± 10.47	< 0.001

Continuous data are expressed as mean ± SD or median (interquartile range). Categorical data are expressed as n (%)

One-way ANOVA, Kruskall Wallis test or Chi-Squared Test

*ALT* alanine aminotransferase; *AST* aspartate aminotransferase; *BMI* body mass index; *BRI* body roundness index; *DBP* diastolic blood pressure; *FBG* fasting blood glucose; *GGT* glutamyl transpeptidase; *HbA1c* hemoglobin A1c; *HDL-C* high-density lipoprotein cholesterol; *SBP* systolic blood pressure; *TC* total cholesterol; *TG* triglyceride; *WC* waist circumference

### Relationship between BRI levels and incident NAFLD during follow-up

Univariate Cox proportional hazards analysis were performed to compare the role of BRI and other variables in predicting T2DM (Additional file 1: Table S1). Incident T2DM was positively associated with age, gender, BMI, BRI, ABSI, WC, ethanol consumption, current-smoke, AST, ALT, GGT, FPG, HbA1c, TG, TC, SBP, and DBP. On the contrary, exercise was irrelevant to T2DM. HDL-C was negatively associated with incident T2DM (Additional file 1: Table S1).

**Fig. 4:**
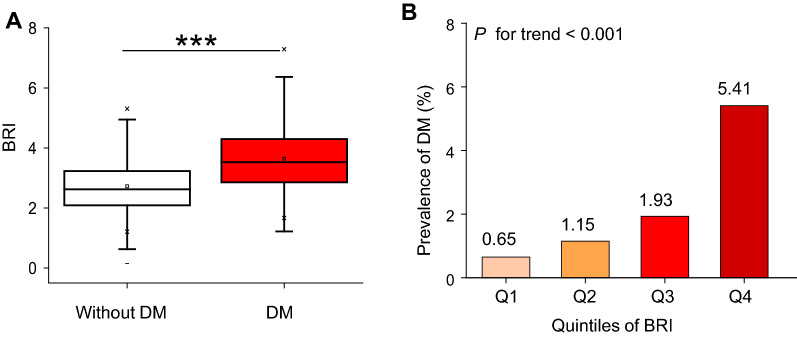
Elevation of BRI and its relationship with incident DM. **A** Levels of BRI in baseline were determined in patients with and without DM. Horizontal lines represent the median values and interquartile ranges. Kruskal–Wallis test. ***P < 0.001. **B** Prevalence of DM stratified according to BRI categories. Linear regression analysis, *P* for trend < 0.001

As shown in Fig. 4A, participants with T2DM had an elevated BRI level [median (interquartile range); 2.624 (2.088–3.231)], as compared with no T2DM [median (interquartile range); 3.526 (2.855–4.300)]. Compared with the lowest quartile in the BRI group, participants with higher quartiles had a higher cumulative incidence [Q1: 0.651(0.398–0.908), Q2: 1.112(0.792–1.452), Q3: 1.933(1.497–2.370), Q4: 5.407(4.690–6.124) *P* < 0.001 for trend] (Table 2 and Fig. 4B).

**Table 2:** The incidence rate of diabetes

BRI	Participants (n)	DM events (n)	Cumulative incidence (95% CI) (%)	Per 100,000 person-year
Total	15,310	350	2.286 (2.049–2.523)	377.304
Q1	3828	25	0.651 (0.398–0.908)	103.434
Q2	3827	44	1.112 (0.792–1.452)	193.004
Q3	3827	74	1.933 (1.497–2.370)	317.249
Q4	3828	207	5.407 (4.690–6.124)	921.214
*P* for trend			< 0.001	

*BRI* body roundness index; *DM* diabetes mellitus; *n* number; *Q* quarter

Kaplan–Meier survival curves for the probability of diabetes-free survival stratified by BRI groups were shown in Fig. 4. A higher BRI level at baseline was associated with a significantly lower probability of diabetes-free survival, indicating the top group with the highest diabetes risk.

The Cox proportional hazards regression model show the association between BRI and incident T2DM. In the crude model, we found that BRI was positively associated with incident T2DM (HR = 2.662, 95% CI 2.377–2.981). This association of BRI and T2DM persisted despite adjustment for sex, age, DBP, SBP, ethanol consumption, smoking status, exercise (HR = 2.285, 95% CI 2.013–2.595) (model I) or inclusion of the baseline mentioned above characteristics and ALT, AST, FBG, HbA1c, HDL-C, TC, TG (model II) (HR = 1.570, 95%CI 1.360–1.811) (Table 3).

**Table 3:** Relationship between BRI and the incident DM in different models

Variable	Crude model	Model I	Model II	GAM
	(HR, 95%CI, *P*)	(HR, 95% CI, *P*)	(HR, 95% CI, *P*)	(HR, 95% CI, *P*)
BRI	2.662 (2.377, 2.981) < 0.001	2.285 (2.013, 2.595) < 0.001	1.570 (1.360, 1.811) < 0.001	1.555 (1.339, 1.806) < 0.001
BRI (quartile)				
Q1	Ref.	Ref.	Ref.	Ref.
Q2	1.946 (1.191, 3.180) 0.007	1.569 (0.957, 2.572) 0.074	1.162 (0.704, 1.918) 0.557	1.251 (0.748, 2.089) 0.393
Q3	3.192 (2.028, 5.023) < 0.001	2.144 (1.348, 3.412) 0.001	1.057 (0.652, 1.713) 0.822	1.070 (0.650, 1.762) 0.789
Q4	9.385 (6.196, 14.216) < 0.001	5.421 (3.504, 8.387) < 0.001	1.892 (1.187, 3.017) 0.007	1.867 (1.150, 3.033) 0.011
*P* for trend	< 0.001	< 0.001	< 0.001	< 0.001

Crude model: we did not adjust other covariants

Model I: we adjust age, gender, SBP, DBP, smoking status, ethanol consumption, habit of exercise

Model II: we adjust age, gender, SBP, DBP, smoking status, ethanol consumption, habit of exercise, ALT, AST, FBG, HbA1c, HDL-c, TC, TG

GAM: All covariates listed in Model II were adjusted. However, continuous covariates were adjusted as non-linearity

*ALT* alanine aminotransferase; *AST* aspartate aminotransferase; *CI* confidence interval; *DBP* diastolic blood pressure; *FBG* fasting blood glucose; *HbA1c* hemoglobin A1c; *HDL-C* high-density lipoprotein cholesterol; *HR* hazard ratio; *Ref* reference; *SBP* systolic blood pressure; *TC* total cholesterol; *TG* triglyceride

### Sensitivity analysis

**Fig. 5:**
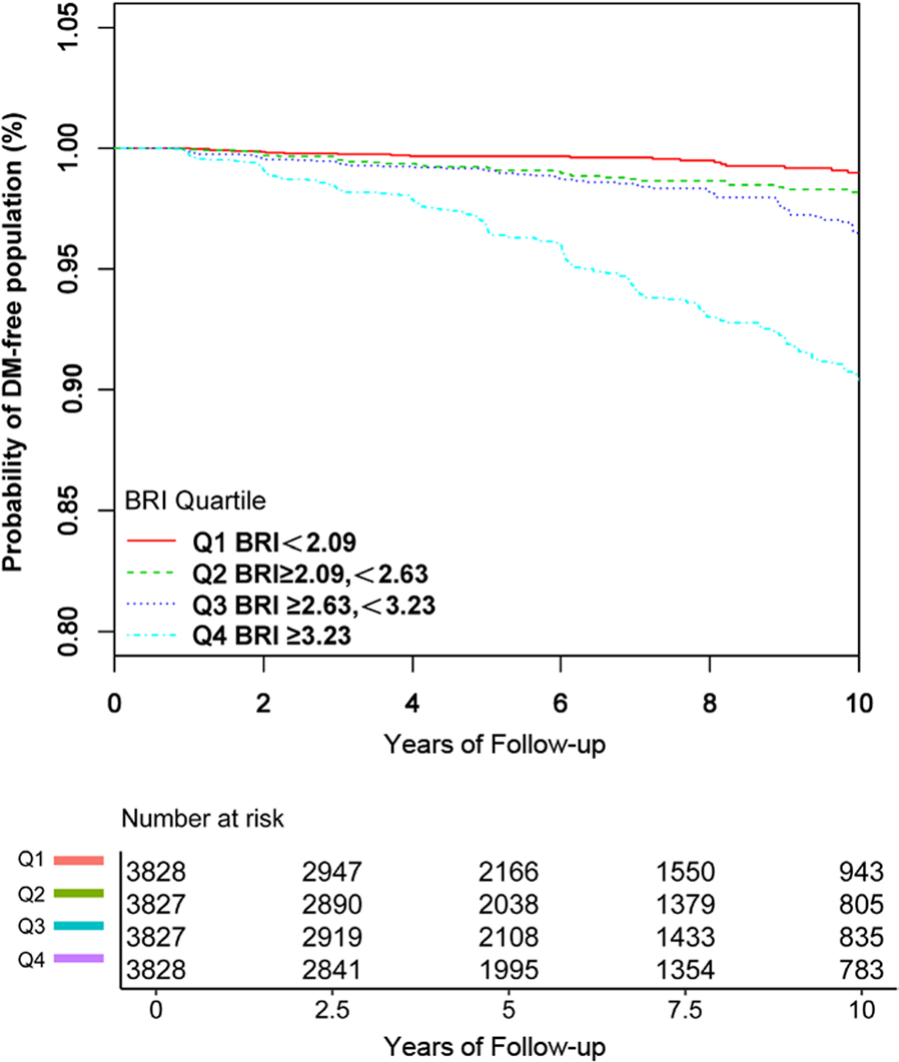
Kaplan Meier curves of overall survival stratified by the following baseline BRI categories

The participants were stratified by the quartiles of the BRI. The trend of T2DM risk in BRI quartiles was significant (*P* for trend < 0.001) (Table 2). Compared with the bottom BRI quartile in the full model, the top quartile had a 89.2 percent increase in T2DM risk with the fully adjusted model (HR = 1.892, 95% CI 1.187–3.017, *P* < 0.001) (Table 3 and Fig. 5). GAM was used to insert the continuity covariate as a curve into the equation, which is basically consistent with Model II with the fully adjusted model (HR = 1.555, 95% CI 1.339–1.806, *P* < 0.001), which proves the robustness of the results (Table 3).

Furthermore, we excluded participants with fatty liver in other sensitivity analyses. The results suggested that BRI was also positively associated with incident T2DM with the fully adjusted model (HR = 1.102, 95% CI 0.867–1.401, *P* = 0.856). We also excluded any alcohol consumers for sensitivity analyses. The results suggested that BRI was still positively associated with incident T2DM with the fully adjusted model (HR = 1.716, 95% CI 1.452–2.027, *P* < 0.001) (Additional file 1: Table S2). The sensitivity analysis results showed that the relationship between BRI and the risk of T2DM was very robust.

### The non-linear relationship between BRI levels and incident NAFLD

**Fig. 6:**
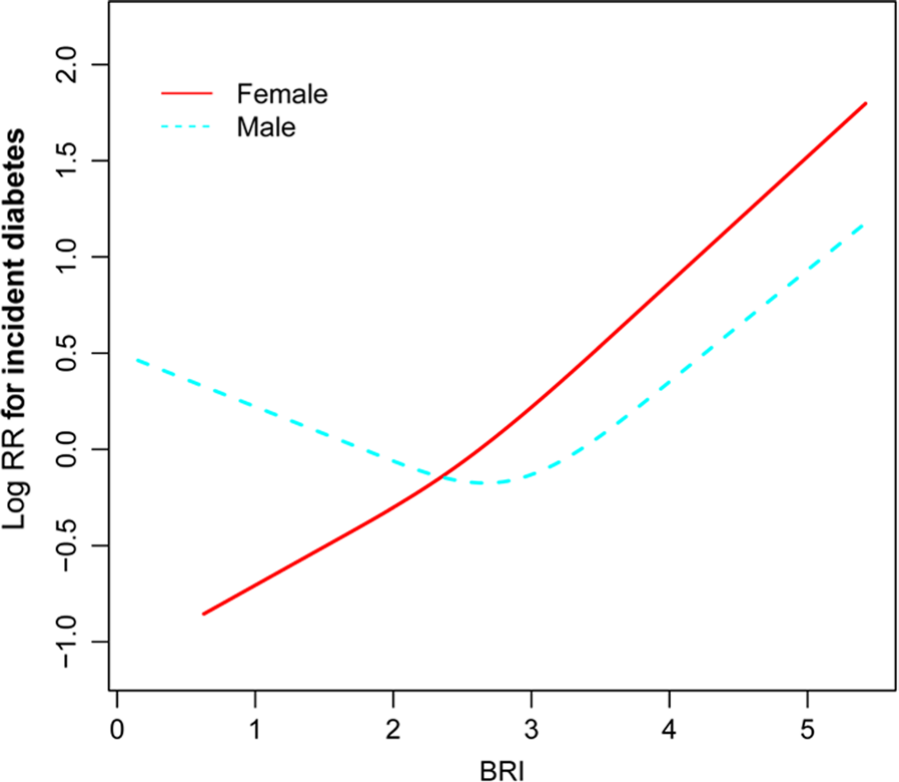
Association of BRI with the risk of DM stratified by sex. The non-linear relationship between BRI and incident of DM. A non-linear relationship between them was detected after adjusting for age, gender, BMI, SBP, DBP, TC, TG, HDL-C, ALT, AST, FBG,HbA1c%, smoking status, ethanol consumption, habit of exercise

GAM and smooth curve fitting were used to study the relationship between BRI levels and incidence of T2DM. A non-linear relationship between the BRI level and the incidence of T2DM was detected after adjusting the confounding variables (age, gender, SBP, DBP, smoking status, ethanol consumption, habit of exercise, ALT, AST, GGT, FBG, HbA1c, HDL-C, TC, TG) (Fig. 6). A binary linear regression model and a recursive algorithm were used to calculate the inflection point of BRI (log likelihood ratio test *P* < 0.001). In male group, the relationship between BRI and T2DM was J-shaped. The inflection point of BRI were 3.147. On the right of inflection point, the effect size was 1.827 (95% CI 1.449–2.303; *P* < 0.001). However, on the left side of the inflection point, we did not observe a significant association between BRI and DM (HR = 0.903, 95% CI 0.606–1.346; *P* = 0.617). In female group, the relationship between BRI and diabetes is close to linear. The inflection point of BRI were 4.137. We found a strong positive correlation between BRI and the incidence of T2DM on the right of the inflection point (Female group: HR = 4.189, 95% CI 1.862–9.421, *P* < 0.001). While on the left side of the inflection point, their association tended to be weakened (Female group: HR = 1.418, 95% CI 0. 983–2.045, *P* = 0.062) (Table 4).

**Table 4:** The result of two-piecewise linear regression model stratified by sex

Incident DM (male)	HR (95% CI)	*P*
Fitting model by standard linear regression	1.475 (1.242, 1.753)	< 0.001
Fitting model by two-piecewise linear regression		
Inflection point of BRI		
≤ 3.146	0.903 (0.606, 1.346)	0.617
> 3.146	1.827 (1.449, 2.303)	< 0.001
*P* for log likelihood ratio test	0.011	

Incident DM (female)	HR (95% CI)	*P*
Fitting model by standard linear regression	1.828 (1.386, 2.412)	< 0.001
Fitting model by two-piecewise linear regression		
Inflection point of BRI		
≤ 4.137	1.418 (0.983, 2.045)	0.062
> 4.137	4.189 (1.862, 9.421)	< 0.001
*P* for log likelihood ratio test	0.045	

We adjusted age, gender, SBP, DBP, smoking status, ethanol consumption, habit of exercise, ALT, AST, GGT, FBG, HbA1c, HDL-c, TC, TG

*ALT* alanine aminotransferase; *AST* aspartate aminotransferase; *CI* confidence interval; *DBP* diastolic blood pressure; *FBG* fasting blood glucose; *GGT* glutamyl transpeptidase; *HbA1c* hemoglobin A1c; *HDL-C* high-density lipoprotein cholesterol; *HR* hazard ratio; *Ref* reference; *SBP* systolic blood pressure; *TC* total cholesterol; *TG* triglyceride

### Relationship between BRI levels and incident T2DM in subgroup analyses

We performed sensitivity analyses to determine whether gender, SBP, DBP, smoking status, alcohol consumption, habit of exercise, HDL-C values, ALT values influenced the relationship between the BRI level and the incident T2DM. Table 5 showed that only alcohol consumption modify the relationship between BRI and incident T2DM (*P* for interaction = 0.0487). And a stronger association was observed in the population without alcohol consumption(HR 1.694; 95% CI 1.435–1.999). In contrast, population with alcohol consumption impared the relationship between BRI and incident T2DM. These results suggested that the relationship between BRI and incident T2DM was robust in most subgroups (Table 5).

**Table 5:** Effect size of BRI on DM in prespecified and exploratory subgroups

Characteristic	No. of participants	HR (95%CI)	*P* value	*P* for interacion
Gender				0.1092
Female	6946	1.845 (1.461, 2.330)	< 0.001	
Male	8364	1.480 (1.254, 1.747)	< 0.001	
SBP (mmHg)				0.9664
< 140	14,569	1.591 (1.375, 1.842)	< 0.001	
≥ 140	741	1.576 (1.024, 2.424)	0.0386	
DBP (mmHg)				0.2317
< 90	14,581	1.639 (1.411, 1.904)	< 0.001	
≥ 90	729	1.239 (0.801, 1.915)	0.3357	
Smoking status				0.089
Never-smoker	8928	1.826 (1.478, 2.255)	< 0.001	
Past-smoker	2938	1.196 (0.863, 1.658)	0.2821	
Current-smoker	3444	1.538 (1.234, 1.917)	0.001	
Alcohol consumption				0.0634
< 40	10,775	1.588 (1.318, 1.913) < 0.0001	< 0.001	
≥ 40, < 140	2788	1.074 (0.744, 1.552) 0.7019	0.7019	
≥ 140, < 280	1360	0.879 (0.537, 1.439) 0.6072	0.6072	
≥ 280				
≥ 280	541	1.274 (0.670, 2.423) 0.4606	0.4606	
Habit of exercise				0.1853
No	12,614	1.529 (1.312, 1.781)	< 0.001	
Yes	2696	1.983 (1.387, 2.834)	0.0002	
HDL-c (mmol/L)				0.1966
< 1	1616	1.426 (1.109, 1.833)	0.0056	
≥ 1	13,694	1.721 (1.467, 2.020)	< 0.0001	
ALT (U/L)				0.5719
≤ 40	14,499	1.621 (1.388, 1.893)	< 0.0001	
> 40	811	1.464 (1.056, 2.029)	0.0221	

Above model adjusted age, gender, SBP, DBP, smoking status, ethanol consumption, habit of exercise, ALT, AST, GGT, FBG, HbA1c, HDL-c, TC, TG

In each case, the model is not adjusted for the stratification variable

*ALT* alanine aminotransferase; *AST* aspartate aminotransferase; *CI* confidence interval; *DBP* diastolic blood pressure; *FBG* fasting blood glucose; *GGT* glutamyl transpeptidase; *HbA1c* hemoglobin A1c; *HDL-C* high-density lipoprotein cholesterol; *HR* hazard ratio; *Ref* reference; *SBP* systolic blood pressure; *TC* total cholesterol; *TG* triglyceride

### BRI as a predictor for T2DM

**Fig. 7:**
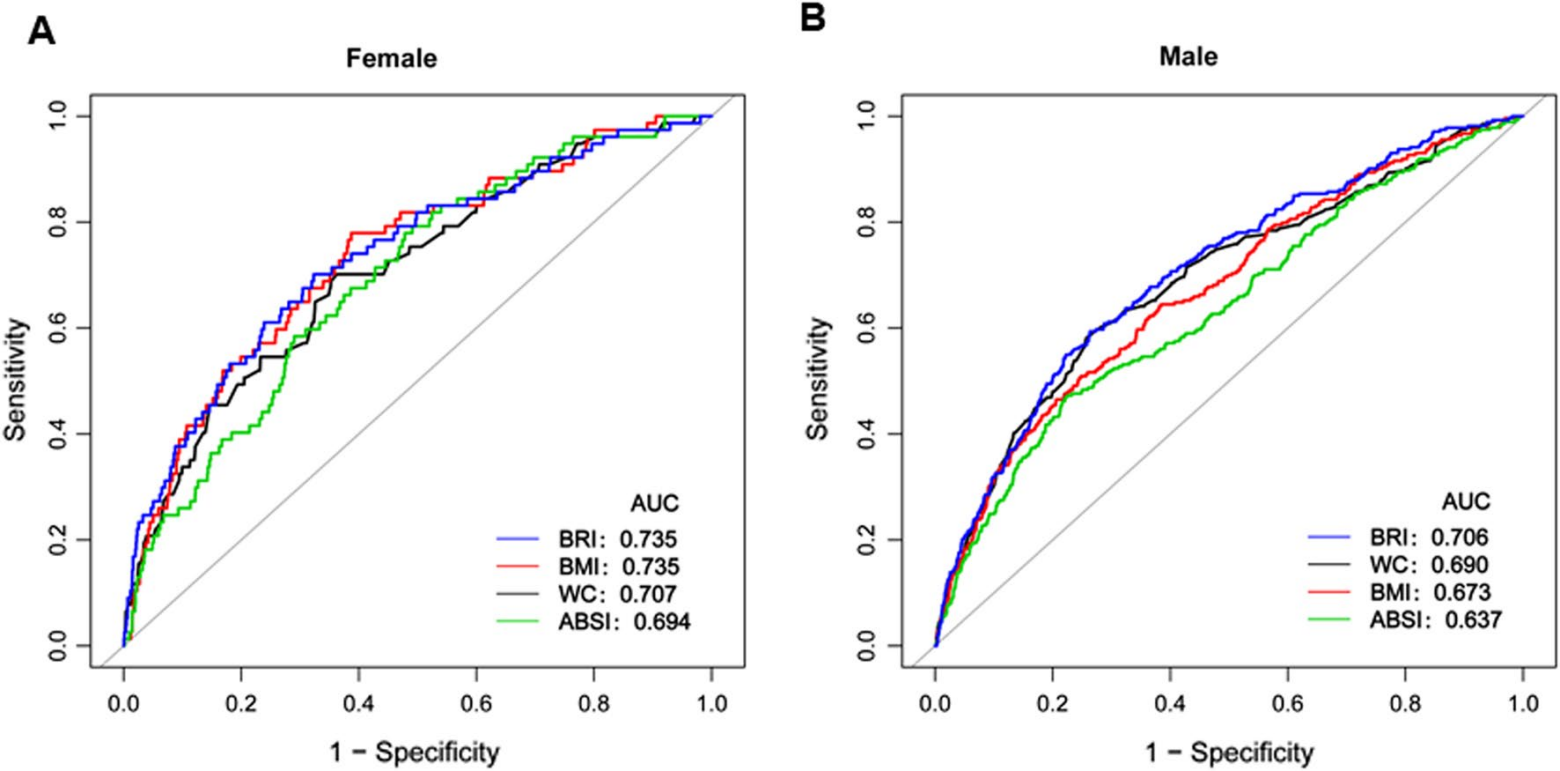
BRI for predicting DM in all participants by ROC analyses stratified by sex

For predicting T2DM, the AUC of BRI were 0.7354 in female and 0.7061 in male (Additional file 1: Table S3). As shown in Fig. 7, the AUC of BRI for predicting T2DM was the greatest among the anthropometric indices, such as BMI, ABSI, and WC. Therefore, BRI value can be used to predicting the incident of T2DM during follow-up.

## Discussion

In this retrospective cohort of 15310 participants, we determined the association between elevated BRI levels and the occurrence of T2DM. The highest BRI level at baseline was associated with the incidence of T2DM after adjusting for age, gender, SBP, DBP, smoking status, alcohol consumption, exercise habits, GGT, AST, ALT, TC, TG, HDL-C, FBG, and HbA1c. In this study, the BRI level has a non-linear relationship with the incidence of T2DM stratified by sex. The inflection points of BRI were 3.146 in male and 4.137 in female. In addition, in male group, the relationship between BRI and T2DM was J-shaped. We found a strong positive correlation between BRI and the incidence of diabetes on the right of the inflection point (Male: HR = 1.827, 95% CI 1.449–2.303; Female: HR = 4.189, 95% CI 1.862–9.421).What’s more, the AUC of BRI for predicting T2DM was the greatest among the anthropometric indices, such as ABSI, WC, and BMI. These data indicate that BRI, as a new anthropometric index, can predict the occurrence of T2DM and provide important prognostic information.

The number of people with diabetes and impaired glucose tolerance has been increasing globally in recent decades [1]. Diabetes brings enormous economic pressure on national health systems around the world, so prevention efforts are needed to reduce this burden [2]. Obesity was considered an major risk factor for the development of T2DM [12]. BMI is an anthropometric measure for diagnosing obesity but it has limitations. It cannot distinguish between muscle-induced and fat-induced weight gain [13]. The concept of BRI provides a more accurate estimate of total percentage of fat and visceral fat tissue.

BRI is a obesity index, which was based on WC and height [5]. Compared with the other anthropometric indices such as WC, and BMI, BRI is a better predictor of body fat and visceral fat tissue volume [14, 15]. Previous studies have examined the association of the anthropometric indicators such as BMI, ABSI, WC, and BRI with T2DM risk and their performance in T2DM risk prediction [16–18]. In obese and overweight Chinese adults, BRI showed the optimal capability to identify IR in the cross-sectional data [14]. In a cross-sectional study, Chang et al. showed that BRI was superior to other anthropometric measures such as BMI, WC, and ABSI in predicting T2DM in Northeast China [16]. In a 15-year prospective study, the discriminatory power of BRI was superior to WC in southwest China [18]. However, the above studies were less in the Japanese population with a longitudinal study. In this longitudinal study of 15310 Japanese participants, a higher cumulative incidence of T2DM was observed at higher baseline BRI levels, which is supported by previous studies showing similar results. The results suggested a significant association between BRI and incident T2DM. BRI (increase by 1) has a significant association with T2DM after adjusting for confounding variables (HR = 1.570, 95% CI 1.360–1.811). The highest quartile of BRI was associated with increased risk for T2DM by 1.892-fold compared with the lowest quartile. Our research compares the AUC of these different anthropometric indicators in the ROC curve in both genders, ROC analysis shows that BRI is the strongest predictor of T2DM, compared with other anthropometric indicators, including BMI, WC, and ABSI in both sex categories.

Chronic ethanol consumption was established as risk factor for T2DM, which can affect glucose metabolism and insulin resistance by impairing pancreatic beta-cell mass and function [19]. In the present study we found alcohol consumption was the risk factors for T2DM. The relationship between ethanol consumption and BRI was linear and positively correlated (data not shown). Ethanol consumption may affect the relationship between BRI and T2DM. However, this study excluded participants with heavy drinking. AND the results showed BRI is an independent risk factor for T2DM. Meanwhile, the sensitivity analysis found that this relationship still exists among participants without alcohol intake.

Previous studies did not explore the possible curvilinear relationship between BRI and T2DM. In the present study, we analyzed the nonlinear relationship between BRI and T2DM for the first time. The result of the smooth curve showed that the relationship between BRI and T2DM was nonlinear in both genders after adjusting for confounders (age, gender, SBP, DBP, smoking status, ethanol consumption, habit of exercise, FBG, HbA1c, AST, GGT, ALT, TC, TG, HDL-C) (*P* < 0.0001). By using a two‐piecewise linear regression model, we calculated the inflection point of BRI. In male group, the relationship between BRI and diabetes is close to linear. When the BRI level was > 3.224, a 1 U/L increase in BRI level is accompanied by a 82.7% increase in HR for T2DM (HR 1.827; 95% CI 1.449–2.303), when the BRI level was ≤ 3.224, the BRI level was no correlation with incident T2DM (HR, 0.903; 95% CI 0.606–1.346) (Table 4). In female group, the relationship between BRI and T2DM was J-shaped. When the BRI level was > 4.137, a 1 U/L increase in BRI level is accompanied by a 318% increase in HR for T2DM (HR 4.189; 95% CI 1.862–9.421), when the BRI level was ≤ 4.137, the BRI level was no correlation with incident T2DM (HR, 1.418; 95% CI 0.983–2.045). Elevated BRI alerts the population at high risk of T2DM during follow-up, which will remind people to adjust their lifestyle habits earlier to improve outcomes.

### Study strength and limitations

The present study has some advantages. First, compared with previous cross-sectional studies, this study was a longitudinal study, which conducted a relatively large sample of the Japanese physical examination population. Second, the non-linear relationship between BRI and T2DM was discovered in this study, and the infection point was calculated at the same time. Third, a series of sensitivity analyses were conducted to ensure the stable of the results, including converting BRI into categorical variables, using GAM to insert continuous covariates into the equation in the form of a curve, and analyze the association between BRI and incident T2DM after excluding alcohol consumers or fatty liver participants. Fourth, perform subgroup analysis to ensure that the relationship between BRI and T2DM is stable among different participants, and the results are stable, and the research results are expected to be successfully promoted in the Japanese physical examination population. In addition, we drew a ROC curve to measure the ability of BRI, BMI, WC, and ABSI to predict the risk of DM.

There are some limitations of the this study. First, our research only focuses on the Japanese population. Therefore, the results of this study cannot be applied to other regions and ethnic groups. Second, participants who had heavy drinking habits, viral hepatitis, or used any drugs at baseline were excluded, so the conclusions in this study may not be applicable to the general population. Third, this retrospective observational study provides the association between BRI and the incident T2DM. Therefore, the results need to be further verified by prospective studies.

## Conclusions

This study demonstrates a positive and non-linear relationship between BRI and incident T2DM in the Japanese population. There was a threshold effect between the BRI level and incident T2DM. When BRI is higher than 4.137 in female and 3.146 in male, it is significant positive correlated with the incident T2DM. The results were expected to provide a reference for clinicians to control BRI. This study shows that BRI can be used as a predictor for early detection and prognosis of T2DM. Abnormal BRI levesl will help clinicians to further identify the high-risk population of T2DM in Japan, which may help clinicians develop management strategies in advance.

## Supplementary Information

**Additional file 1: Table S1.** The results of univariate COX regression. **Table S2.** Relationship between BRI and incident diabetes in different sensitivity analyses. **Table S3.** AUC with the 95% CI of BMI, WC, ABSI and BRI for predicting DM stratified by sex.

## Data Availability

Data can be downloaded from ‘DATADRYAD’ database (www.Datadryad.org).

## Abbreviations

ABSI: A body shape index
ALT: Alanine aminotransferase
AST: Aspartate aminotransferase
AUC: Area under the curve
BRI: Body roundness index
BMI: Body mass index
CI: Confidence interval
DBP: Diastolic blood pressure
FPG: Fasting plasma glucose
GAM: Generalized additive models
GGT: Gammaglutamyltransferase
HbA1c: HemoglobinA1c
HDL-C: High-density lipoprotein cholesterol
ROC: Receiver operating characteristic
SBP: Systolic blood pressure
T2DM: Type 2 diabetes mellitus
TC: Total cholesterol
TG: Triglyceride
WC: Waist circumference
