# Supplementary material for: Non-linear relationship between the body roundness index and incident type 2 diabetes in Japan: a secondary retrospective analysis
Source: J Transl Med. 2022 Mar 7;20:110. doi: 10.1186/s12967-022-03321-x (PMC8900386; doi:10.1186/s12967-022-03321-x)
Supplement: Supplementary file 1 — Additional file 1: Table S1. The results of univariate COX regression. Table S2. Relationship between BRI and incident diabetes in different sensitivity analyses. Table S3. AUC with the 95% CI of BMI, WC, ABSI and BRI for predicting DM stratified by sex. [file 12967_2022_3321_MOESM1_ESM.docx]

Supplementary data

Non-linear relationship between the body roundness index and incident type 2 diabetes in Japan: a secondary retrospective analysis

Running title: body roundness index and incident diabetes mellitus

Liling Wu^1,2†^, Hailu Pu^1,2^, Man Zhang^3,4^, Haofei Hu^1,2*^, Qijun Wan^1,2*^

^1^Department of Nephrology, The First Affiliated Hospital of Shenzhen University, Shenzhen 518000, Guangdong Province, China.

^2^Department of Nephrology, Shenzhen Second People’s Hospital, Shenzhen 518000, Guangdong Province, China.

^3^Department of Functional Neurology, The First Affiliated Hospital of Shenzhen University, Shenzhen 518000, Guangdong Province, China.

^4^Department of Functional Neurology, Shenzhen Second People’s Hospital, Shenzhen 518000, Guangdong Province, China.

Full list of author information is available at the end of the article.

^*^Correspondence: [yiyuan2224@sina.com](mailto:yiyuan2224@sina.com) and huhaofei0319@126.com.

**Total number of tables**: 3

**Table S1. The results of univariate COX regression**

|  | **Statistics** | **HR（95%CI）** | | ***P*-value** | |
| --- | --- | --- | --- | --- | --- |
| Age, years | 43.71 ± 8.90 | 1.06 (1.05, 1.07) | | <0.001 | |
| **Gender -n (%)** | | | <0.001 | |  |
| Female | 6946 (45.37%) | 1.0 | |  | |
| Male | 8364 (54.63%) | 2.71 (2.10, 3.49) | |  | |
| BRI | 2.71 ± 0.83 | 2.66 (2.38, 2.98) | | <0.001 | |
| TC (mmol/L) | 5.12 ± 0.86 | 1.47 (1.32, 1.64) | | <0.001 | |
| TG (mmol/L) | 0.91 ± 0.65 | 1.80 (1.68, 1.93) | | <0.001 | |
| HDL-c (mmol/L) | 1.46 ± 0.40 | 0.15 (0.11, 0.21) | | <0.001 | |
| SBP (mmHg) | 114.30 ± 14.85 | 1.03 (1.02, 1.04) | | <0.001 | |
| DBP (mmHg) | 71.47 ± 10.45 | 1.05 (1.04, 1.06) | | <0.001 | |
| FBG (mmol/L) | 5.16 ± 0.41 | 26.53 (19.34, 36.37)) | | <0.001 | |
| HbA1c（%） | 5.17 ± 0.32 | 56.83 (40.95, 78.87) | | <0.001 | |
| GGT (U/L) | 20.21 ± 18.00 | 1.01 (1.01, 1.01) | | <0.001 | |
| ALT (U/L) | 19.83 ± 14.05 | 1.01 (1.01, 1.01) | | <0.001 | |
| AST (U/L) | 18.33 ± 8.50 | 1.01 (1.01, 1.01) | | <0.001 | |
| Ethanol consumption g/wk | 47.97 ± 82.50 | 1.00 (1.00, 1.00) | | <0.001 | |
| Smoking status | | | | <0.001 | |
| Never-smoker | 8928 (58.31%) | 1.0 | |  | |
| Past-smoker | 2938 (19.19%) | 1.83 (1.38, 2.43) | |  | |
| Current-smoker | 3444 (22.50%) | 2.72 (2.15, 3.45) | |  | |
| Habit of exercise | | | | 0.069 | |
| No | 12614 (82.39%) | 1.0 | |  | |
| Yes | 2696 (17.61%) | 0.75 (0.56, 1.02) | |  | |
| Fatty liver | | | | <0.001 | |
| No | 12688 (82.87%) | 1.0 | |  | |
| Yes | 2622 (17.13%) | 6.67 (5.39, 8.25) | |  | |

ALT: alanine aminotransferase; AST: aspartate aminotransferase; BRI: body roundness index; DBP: diastolic blood pressure; FBG: fasting blood glucose; GGT: glutamyl transpeptidase; HbA1c: hemoglobin A1c; HDL-C: high-density lipoprotein cholesterol; SBP: systolic blood pressure; TC: total cholesterol; TG: triglyceride.

**Table S2. Relationship between BRI and incident diabetes in different sensitivity analyses**

| Exposure | ModelI (HR,95%CI,P) | Model II (HR,95%CI,P) |
| --- | --- | --- |
| BRI | 1.102 (0.867, 1.401) 0.427 | 1.716 (1.452, 2.027) <0.001 |
| BRI (quartile) |  |  |
| Q1 | Ref | Ref |
| Q2 | 1.106 (0.648, 1.887) 0.712 | 1.085 (0.592, 1.990) 0.791 |
| Q3 | 0.838 (0.478, 1.472) 0.539 | 1.304 (0.744, 2.283) 0.353 |
| Q4 | 1.115 (0.637, 1.954) 0.702 | 2.434 (1.415, 4.187) 0.001 |
| *P* for trend | 0.856 | <0.001 |

Model I was sensitivity analysis after excluding those with fatty liver. we adjust age, gender, SBP, DBP,

smoking status, ethanol consumption, habit of exercise, ALT, AST, GGT, FBG, HbA1c, HDL-C, TC, TG

Model II was sensitivity analysis after excluding any alcohol consumers. we adjust age, gender, SBP, DBP,

smoking status, ethanol consumption, habit of exercise, ALT, AST, GGT, FBG, HbA1c, HDL-C, TC, TG

BRI: body roundness index; HR: hazard ratios; CI: confidence interval; Ref: reference.

**Table S1.** The results of univariate COX regression. **Table S2.** Relationship between BRI and incident diabetes in different sensitivity analyses

**Table S3. AUC with the 95% CI of BMI, WC, ABSI and BRI for predicting DM stratified by sex.**

| **Variables** | **AUC** | **95% CI lower bound** | **95% CI upper bound** | **Best threshold** | **Specificity** | **Sensitivity** | **Yorden Index** |
| --- | --- | --- | --- | --- | --- | --- | --- |
| **Male** | | | | | | | |
| BMI | 0.6732 | 0.6395 | 0.7069 | 24.6586 | 0.7521 | 0.5092 | 0.2613 |
| WC | 0.6898 | 0.6556 | 0.7240 | 0.8465 | 0.7421 | 0.5824 | 0.3245 |
| ABSI | 0.6371 | 0.6017 | 0.6724 | 0.0048 | 0.7760 | 0.4725 | 0.2485 |
| BRI | 0.7061 | 0.6740 | 0.7382 | 3.2961 | 0.7367 | 0.5934 | 0.3301 |
| **Female** | | | | | | | |
| BMI | 0.7347 | 0.6774 | 0.7920 | 21.2328 | 0.6130 | 0.7792 | 0.3922 |
| WC | 0.7069 | 0.6465 | 0.7673 | 0.7345 | 0.6375 | 0.7013 | 0.3388 |
| ABSI | 0.6941 | 0.6381 | 0.7501 | 0.0063 | 0.5092 | 0.7922 | 0.3014 |
| BRI | 0.7354 | 0.6755 | 0.7953 | 2.8025 | 0.6768 | 0.7013 | 0.3781 |

ABSI: a body shape index; AUC: area under the curve; BRI: body roundness index; BMI: body mass index; CI: confidence interval;

DM: diabetes mellitus; WC: waist circumference.
